# Supplementary material for: Optimised nanobody-based quenchbodies for enhanced protein detection
Source: Commun Biol. 2025 Jun 18;8:937. doi: 10.1038/s42003-025-08359-3 (PMC12177037; doi:10.1038/s42003-025-08359-3)
Supplement: Supplementary file 2 — Supplementary Material [file 42003_2025_8359_MOESM2_ESM.pdf]

Supplementary Figures

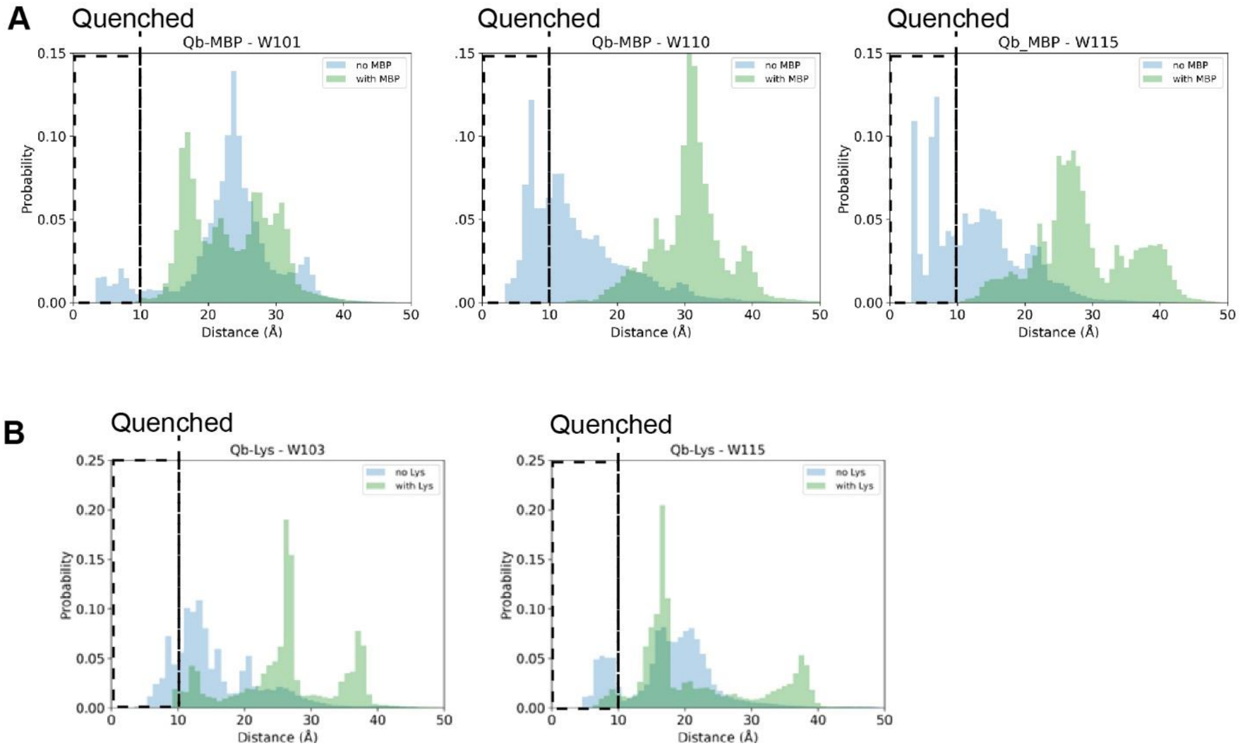

Figure S1. Design and *in silico* modelling of the MBP-quenchbody. Normalised distribution histograms derived from MD simulations illustrating the fluorophore-CDR-tryptophan distances for (A) W101, W110 and W115 in the absence (blue) or presence (green) of antigen for the MBP-binding nanobody (PDB ID: 5M14) and for (B) W103 and W115 in the absence (blue) or presence (green) of antigen for the Lys-binding nanobody (PDB ID: 1ZVH). The fluorophore is considered quenched by tryptophan at distances ≤ 10 Å (hatched zone).

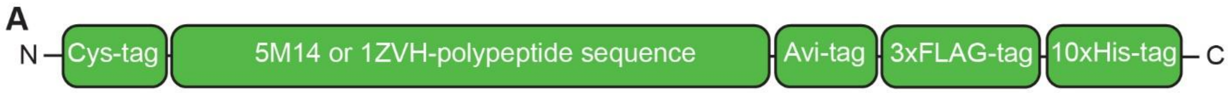

B

>Qb-MBP

MSKQIEVNYCSNETGSQVQLVESGGGSVQAGGSLRLSCAASGQIEHIGYLGWFRQAPGKEREGVAALITY
TGHTYYADSVKGRFTVSLDNAKNTVYLQMNSLKPEDTALYYCAAAEWGSQSPLTQWFYRYWGQGTQVT
VSGSGLNDIFEAQKIEWHEDYKDHDGDYKDHDIDYKDDDDKHHHHHHHHHH

>Qb-Lys

MSKQIEVNYCSNETGSDVQLVESGGGSVQAGGSLRLSCAASGYIASINYLGWFRQAPGKEREGVAAVSPA
GGTPYYADSVKGRFTVSLDNAENTVYLQMNSLKPEDTALYYCAAARQGWYIPLNSYGYNYWGQGTQVT
VSSRGRGSGLNDIFEAQKIEWHEDYKDHDGDYKDHDIDYKDDDDKHHHHHHHHHH

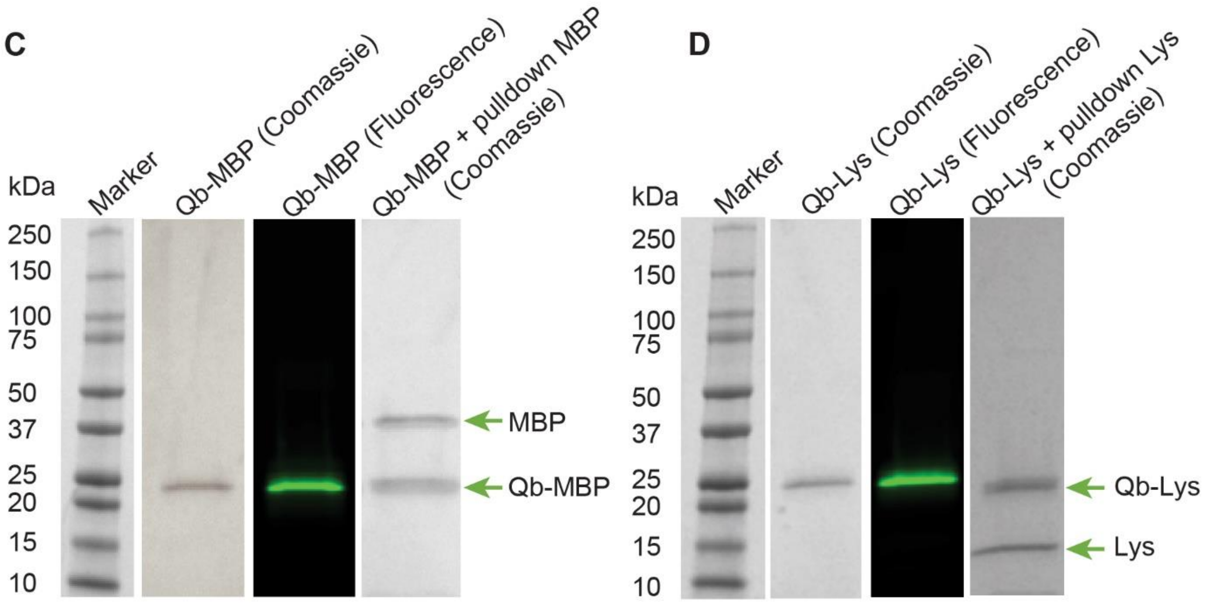

Figure S2. Quenchbody design schematic with reducing SDS-PAGE analysis of quenchbody labelling, purification and functionality (A) Protein coding sequences from either the 5M14 MBP-binding nanobody or the 1ZVH lysozyme-binding nanobody were designed with an N-terminal Cys-tag to facilitate fluorophore labelling and a C-terminal Avi-tag to facilitate biotinylation. Both a 3x FLAG-tag and a 10xHis-tag were also included to facilitate purification procedures. The entire protein coding element was synthesised as part of an IVTT-expression DNA block containing flanking elements for cell-free expression of the quenchbodies.(B) Full peptide sequence of Qb-MBP and Qb-Lys (C) Purified Qb-MBP and (D) purified Qb-Lys were analysed by reducing SDS-PAGE and subjected to in-gel fluorescence imaging to detect TAMRA or stained with Instant Blue to assess protein purity. Quenchbody functionality was further probed by reducing SDS-PAGE analysis of pulldown assays, in which each of the Qbs were immobilised on FLAG beads and incubated with 1 μM cognate antigen prior to extensive washing and FLAG elution.

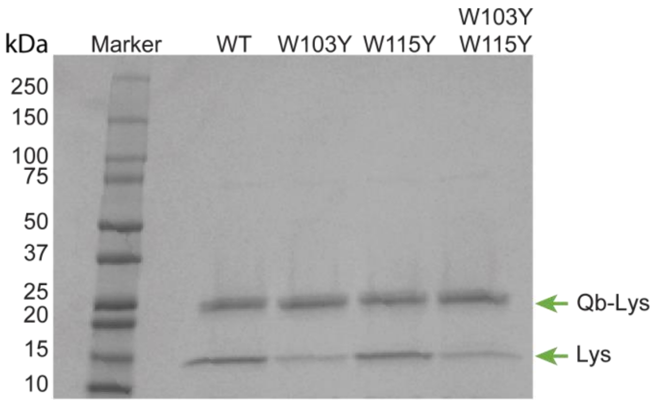

Figure S3. Lysozyme pulldown by Qb-Lys and tryptophan substitution mutants. The relative binding capacity of W103Y, W115Y and W103Y/W115Y for lysozyme was compared to the WT using a pulldown assay and analysed by reducing SDS-PAGE, showing that the W103Y substitution impacts the binding for lysozyme, but not W115Y. Note Qb-Lys-WT lane appears in Figure S1D to demonstrate pulldown.

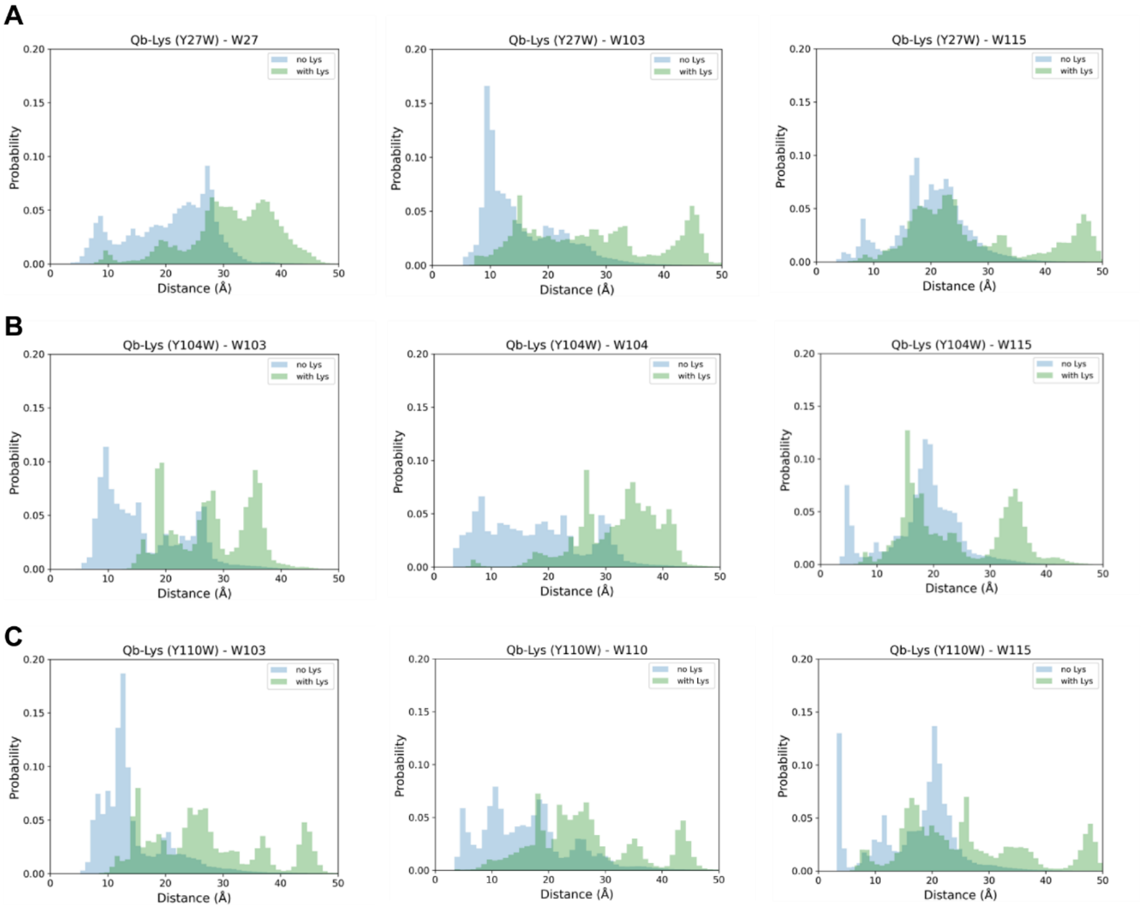

Figure S4. *In silico* modelling of the Lys-quenchbody Y27W, Y104W and Y110W mutants. Normalised distribution histograms illustrating the TAMRA-tryptophan distances derived from MD simulations in the absence (blue) or presence (green) of lysozyme. TAMRA is considered quenched at distances ≤ 10 Å.

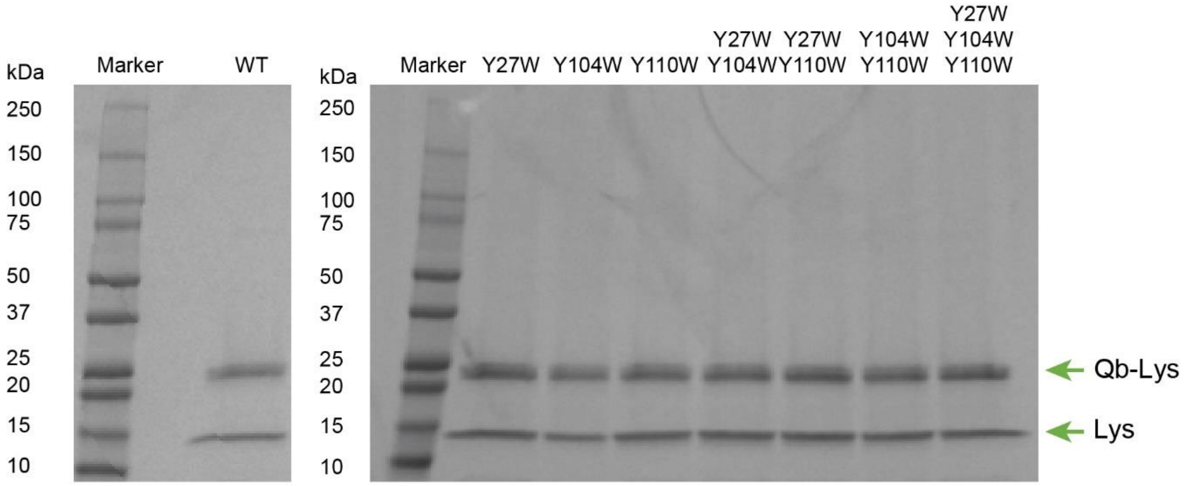

Figure S5. **The relative binding capacity of lysozyme-binding quenchbody mutants** Y27W, Y104W, Y110W, Y27W/Y104W Y27W/Y110W, Y104W/Y110W and Y27W/Y104W/Y110W for lysozyme was assessed using a pulldown assay and analysed by reducing SDS-PAGE. Comparison to WT control from Fig S3.

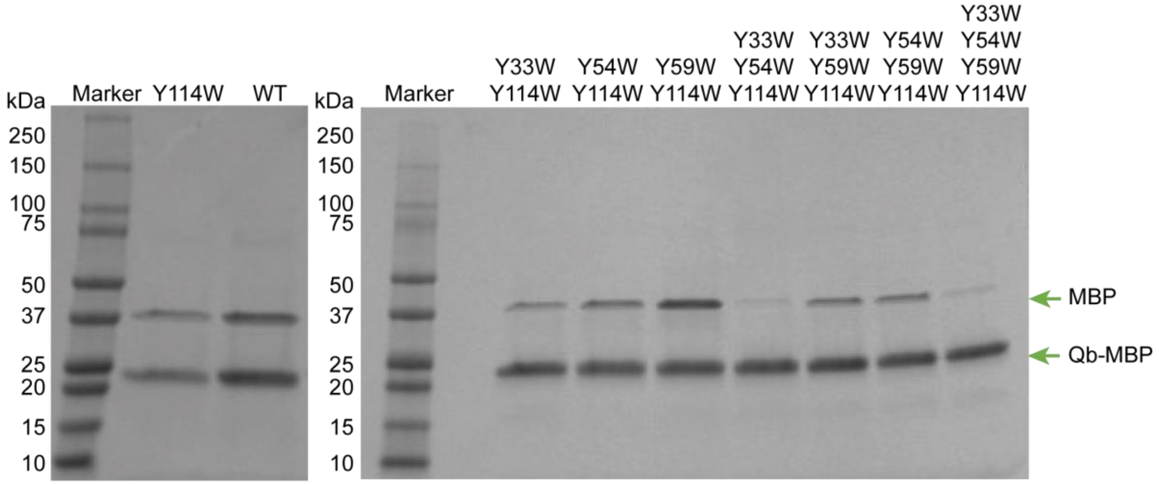

Figure S6. MBP pulldown by Qb-MBP and tryptophan substitution mutants. The relative binding capacity of Qb-MBP and variants Y114W, Y33W/Y114W, Y54W/Y114W, Y59W/Y114W, Y33W/Y54W/Y114W, Y33W/Y59W/Y114W, Y54W/Y59W/Y114W, Y33W/Y54W/Y59W/Y114W was assessed using a pulldown assay and analysed by reducing SDS-PAGE.

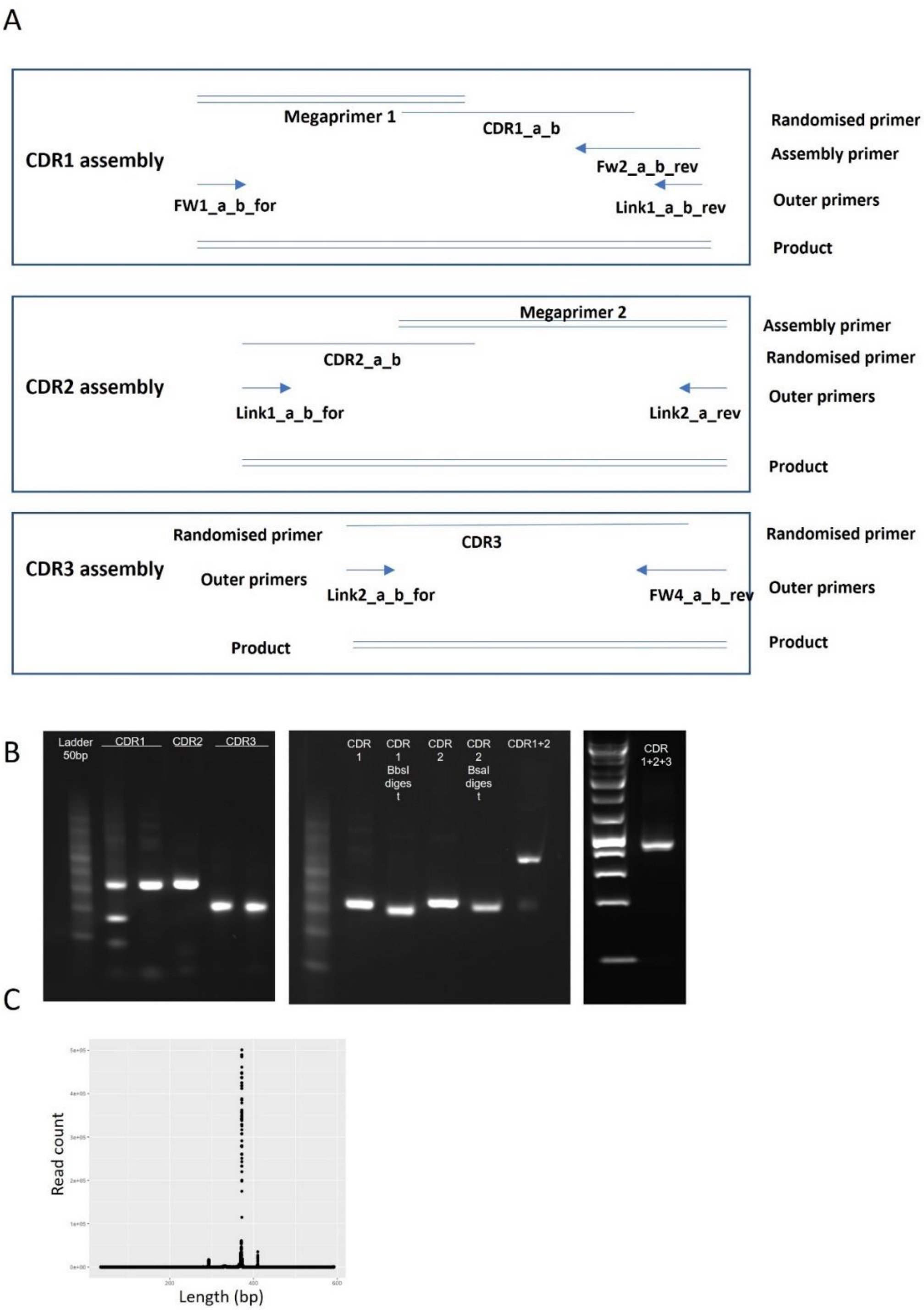

Figure S7. Assembly of quenchbody libraries. A) Schematic shows the polymerase mediated assembly of CDR1-3 with associated framework sequences. B) DNA gel electrophoresis images of CDR assemblies (left), digested CDR1 and 2, as well as the ligation product of CDR1+2 (middle) and the completed assembly (right). C) Histogram of assembled read lengths following NGS validation of assembled quenchbody library.

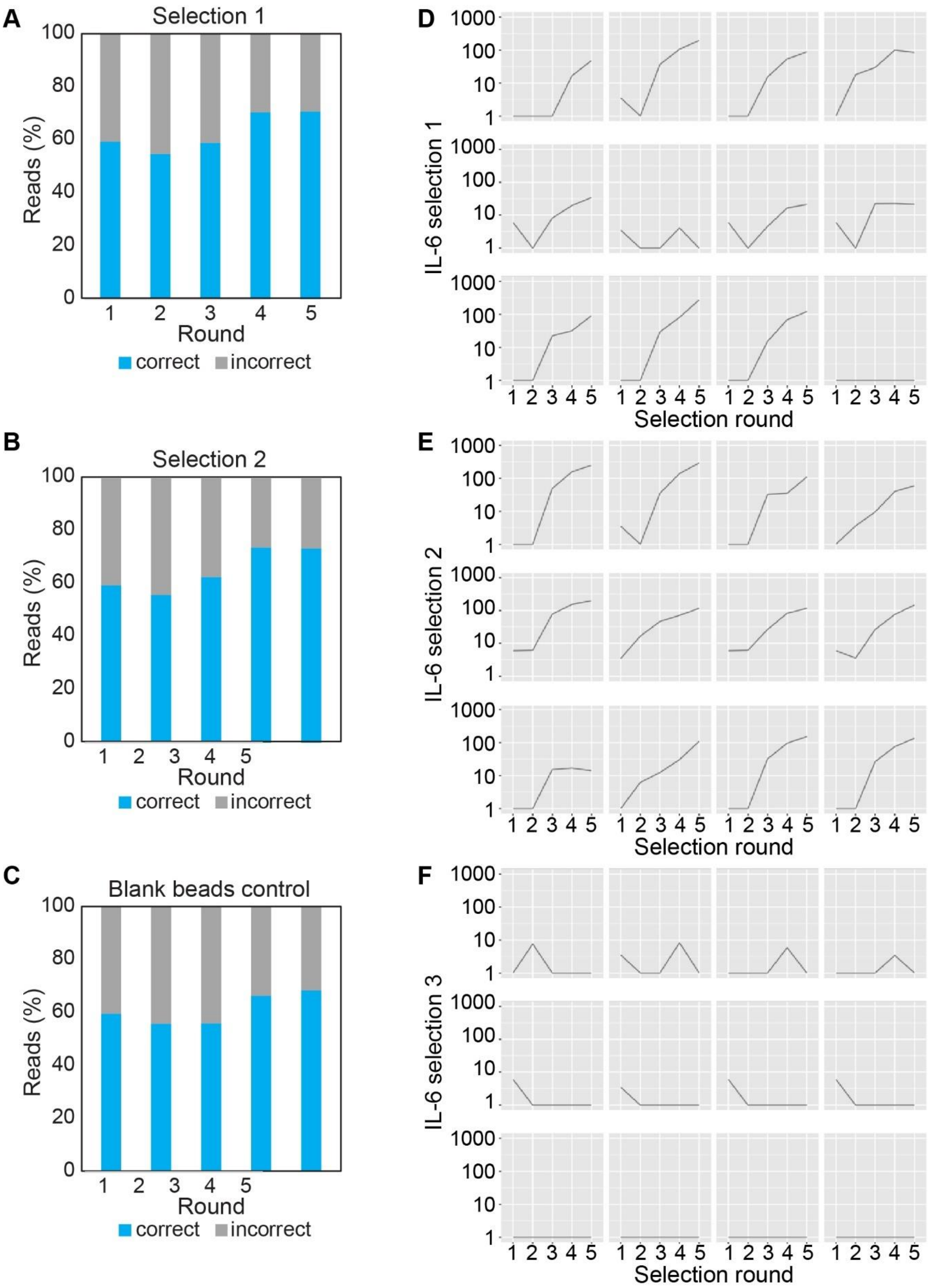

Figure S8. Additional data on SNAP-display selections. (A-C) Fractions of assembled quenchbody NGS reads over rounds 1 – 5 for IL-6 replicates 1, 2 and blank beads, respectively. (D-F) Enrichment curves of individual quenchbody hits in each selection.

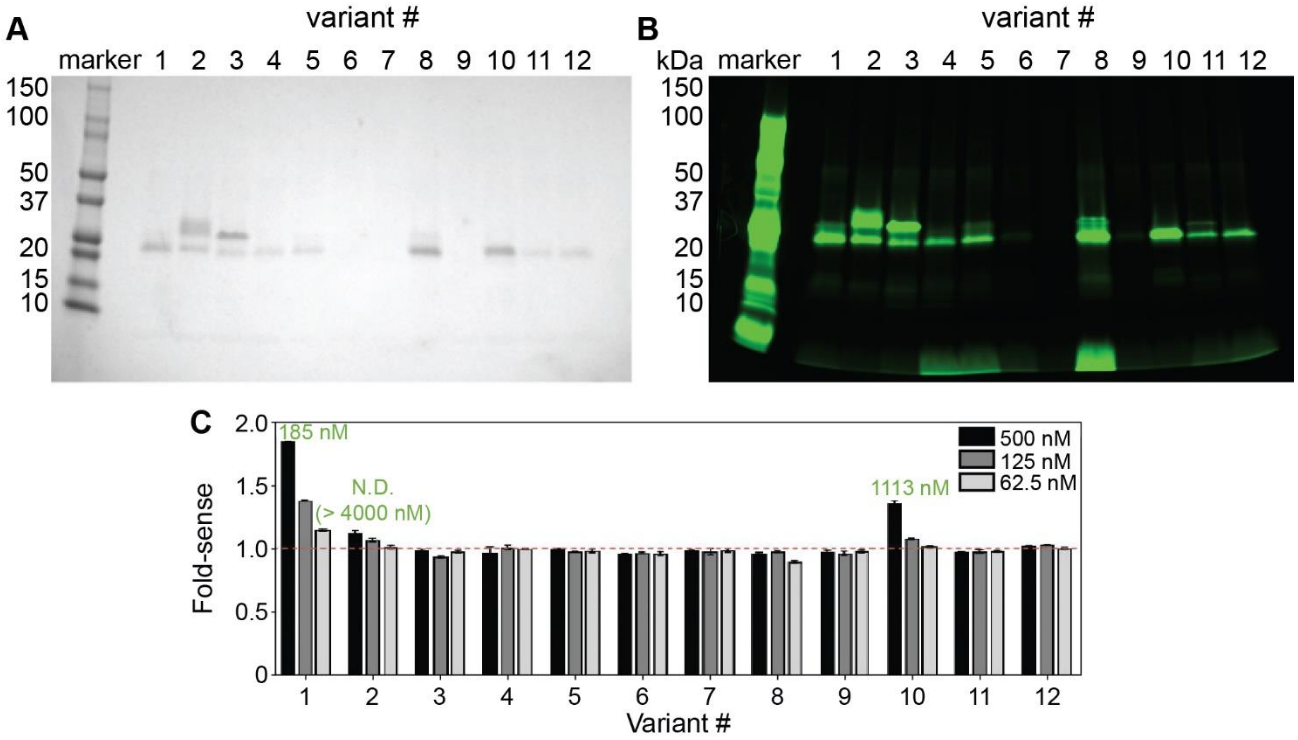

Figure S9. Production of the top twelve hits as quenchbodies. Reducing SDS-PAGE (A) following staining with Coomassie blue to assess protein purity, and (B) in-gel fluorescence imaging to detect TAMRA. A pre-screen with 500 nM IL-6 showed that Qb-IL6-1 and Qb-IL6-10 had the highest fluorescence upon IL6 binding. (C) Relative fluorescent intensity changes in TAMRA-labelled IL6 quenchbodies upon antigen binding compared to quenchbody alone (fold-sense). Quenchbodies selected against IL-6 (Qb-IL6-1, 2, 3, 4, 5, 6, 7, 8, 9, 10, 11, or 12) were incubated (60 min, 25°C) in the presence of 500, 125 or 62.5 nM IL-6. The EC_{50} as a proxy measure for quenchbody binding affinity (K_D) is displayed on top of the bars for responding variants. Data are mean ± SD (n = 3) normalised fluorescence intensity.

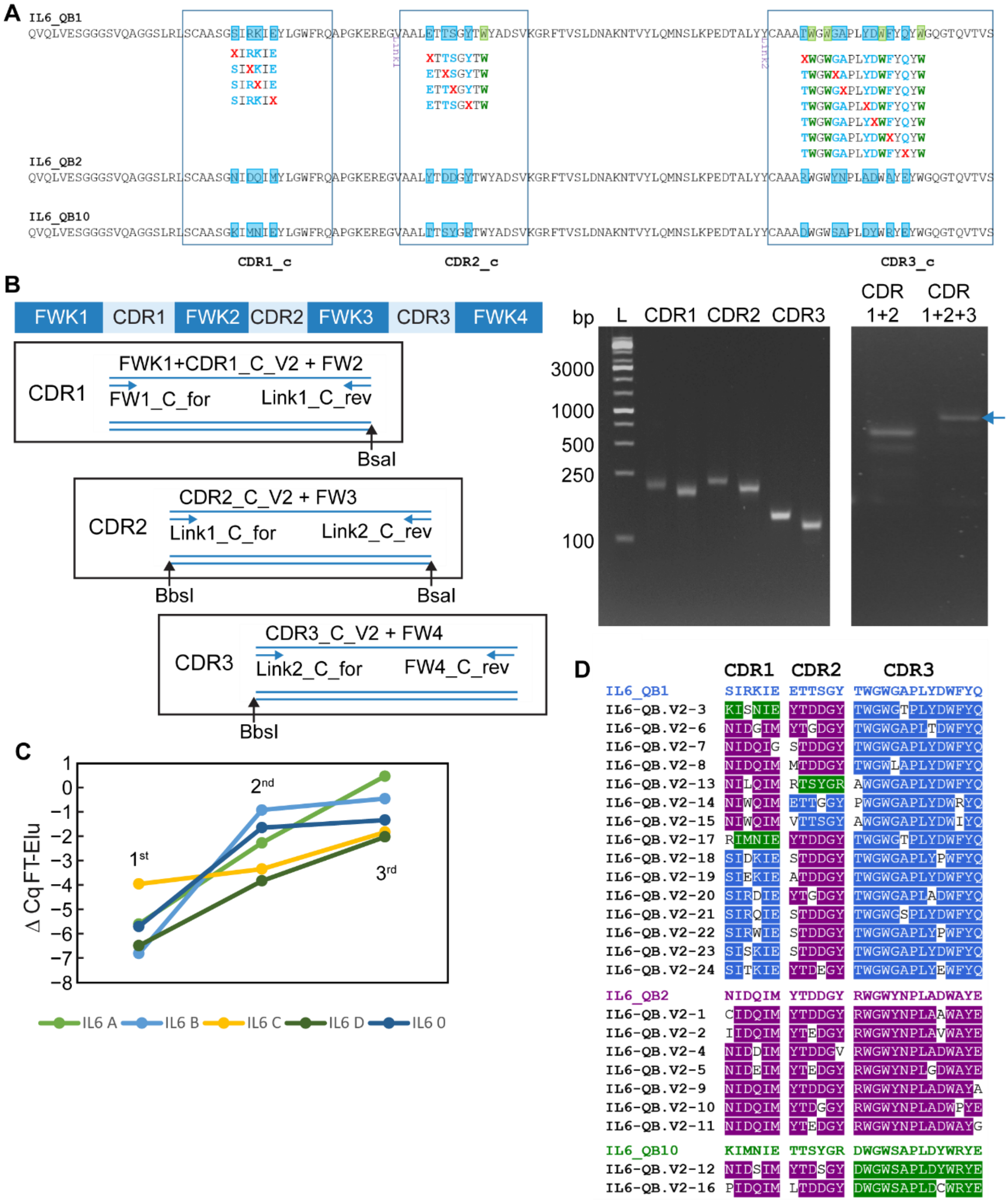

Figure S10. Library design of IL6 quenchbody for affinity maturation. (A) Tryptophan residues are indicated in green. Mutation positions are indicated in blue and NNK codons are indicated in red. (B) Library assembly using golden gate enzymes (BsaI and BbsI), FWK: Framework. CDR: complimentary-determining region. (C) Enrichment of SNAP gene over rounds 1–3 measured by qPCR. Data points are delta Cq values of the flowthrough (FT) and elution (Elu). (D) Clustering of the affinity maturation hits to the three IL-6 parent quenchbodies according to the the three CDRs. Hits associated with IL6-QB1, IL6-QB2 and IL6-QB10 are indicated in blue, purple and green respectively.

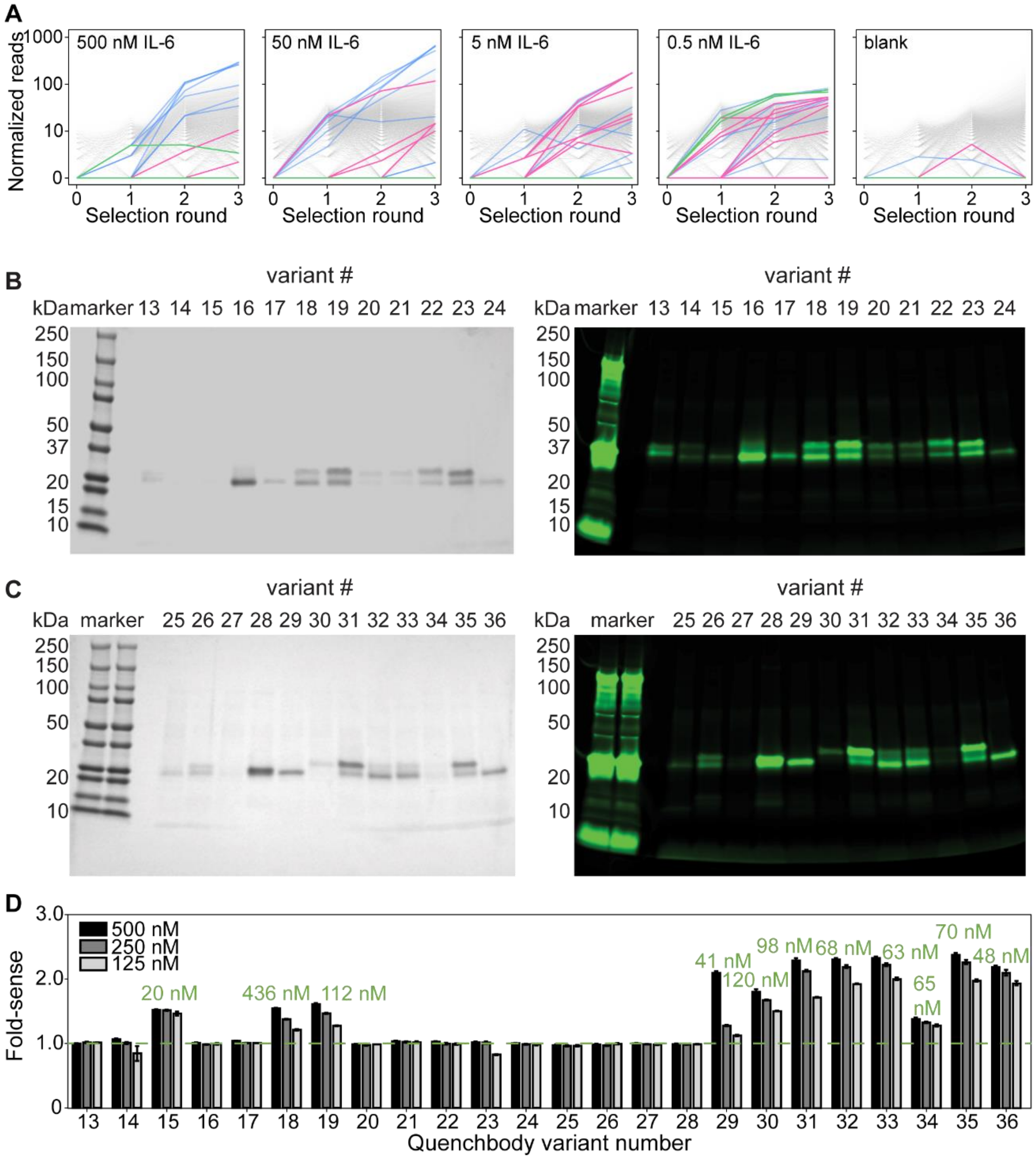

Figure S11. Production and characterisation of affinity matured IL-6 binding quenchbodies. (A) Enrichment curves of affinity maturation for quenchbody hits QB1, QB2 and QB3 combinatorial library. Lines show normalised read counts over rounds 1–3 for selection against of IL-6 (concentration A = 500 nM, B = 50 nM, C = 5 nM, D = 0.5 nM), or blank beads (0 nM). Qb-IL6-1 = blue, Qb-IL6-2 = magenta and Qb-IL6-10 = green. Reducing SDS-PAGE of purified quenchbodies variants 13–24 (B) and 25–36 (C) with Instant Blue staining (left) and in-gel TAMRA fluorescence (green) of quenchbodies showing successful labelling of quenchbodies with varying degrees of free TAMRA contamination that appears to be quenchbody dependent, despite extensive washing. (D) Relative fluorescent intensity changes in TAMRA-labelled IL6 quenchbodies upon antigen binding compared to quenchbody alone (fold-sense). Quenchbodies selected against IL-6 (Qb-IL6-13–36) were incubated (60 min, 25°C) in the presence of 500, 250 or 125 nM IL-6. The EC_{50} as a proxy measure for quenchbody binding affinity (K_D) is displayed on top of the bars for responding variants. Data are mean ± SD (n = 3) normalised fluorescence intensity.

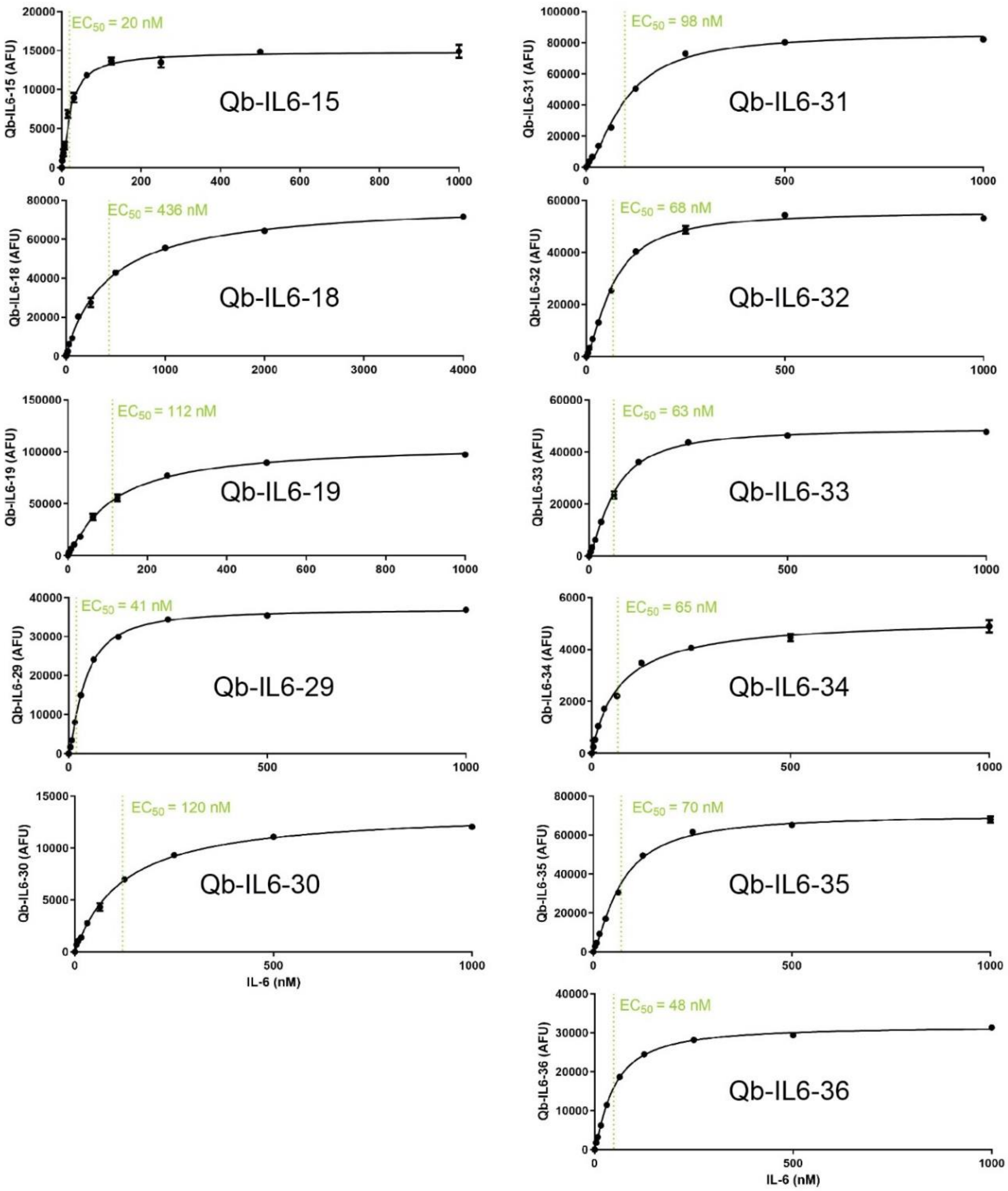

Fig. S12. Fluorescence intensity changes in TAMRA-labelled IL-6 quenchbodies upon antigen binding. Fluorescence intensity responses of Qb-IL6-15, 18, 19, 29, 30, 31, 32, 33, 34, 35 and 36 were analysed in a CLARIOstar fluorescence plate assay and fit to an equation describing a single site-specific binding mode to derive an EC_{50} as a proxy measure for quenchbody binding affinity (KD). Data are mean ± SD normalised fluorescence intensity (n = 3) by subtracting the fluorescence (AFU) of the 0 nM antigen control from all samples.

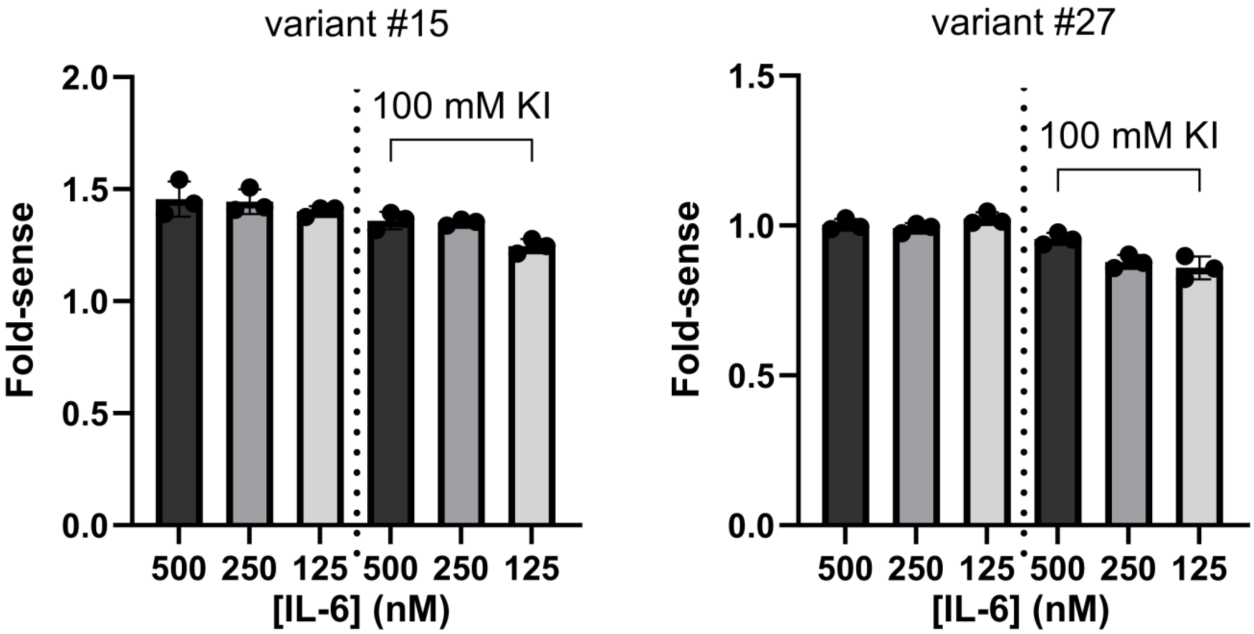

Figure S13. Effect of potassium iodide as an in-solution quencher for improving response of low-responder and null-responding IL-6 quenchbodies. Relative fluorescent intensity changes in TAMRA-labelled IL6 quenchbody variant #15 (low responder) and variant #27 (null-responder) in the absence or presence of 100 mM potassium iodide upon antigen binding compared to quenchbody alone (fold-sense). Quenchbodies were incubated (60 min, 25°C) in the presence of 500, 250 or 125 nM IL-6. Data are mean ± SD (n = 3) normalised fluorescence intensity.

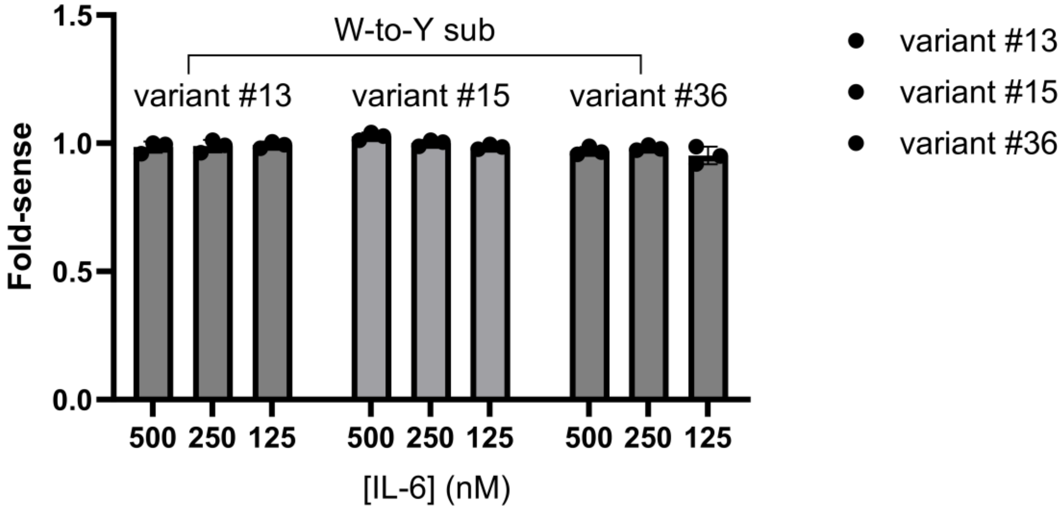

Figure S14. Effect of W-to-Y substitution on fluorescence response of IL-6 quenchbody variants in the presence of antigen. Relative fluorescent intensity changes in TAMRA-labelled IL6 quenchbody variants #13, #15 and #36 with favourable sensing tryptophans removed by substituion with tyrosines (W59Y, W101Y, W103Y, W110Y, and W115Y). Variants were incubated (60 min, 25°C) in the presence of 500, 250 or 125 nM IL-6. Data are mean ± SD (n = 3) normalised fluorescence intensity.

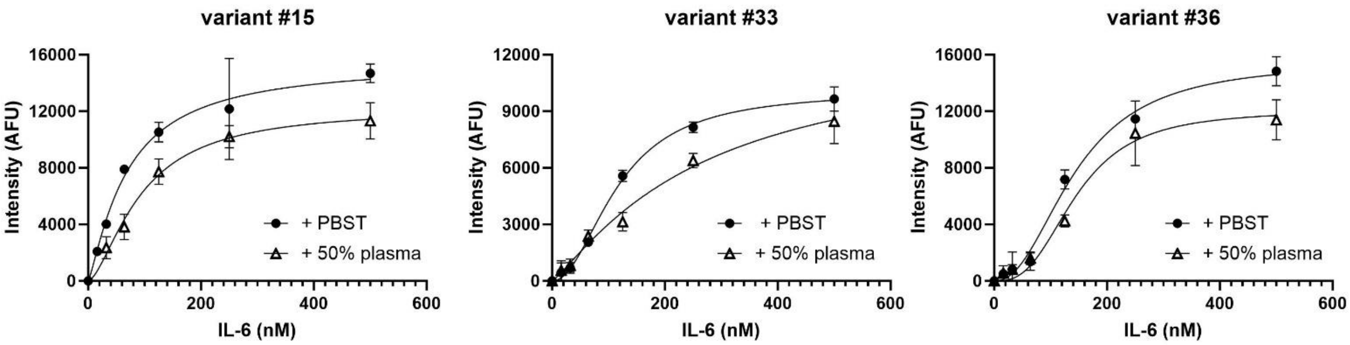

Figure S15. Fluorescence intensity changes in TAMRA-labelled IL-6 quenchbodies upon antigen binding following spiking with 50% human plasma. Biotinylated quenchbodies were added to a neutravidin-coated plate with 0 – 500 nM IL-6 in PBST or 50% human plasma. After incubation for 30 min which captured quenchbody-IL-6 complexes to the bottom of the plate, PBST and 50% human supernatants were removed and PBST was added to all wells for measuring of fluorescence intensity, without washing. Data are fluorescent intensity (AFU) blank corrected against quenchbody in the absence of IL-6 (n = 2).

Supplementary Tables

Table S1 Percentage time derived from MD simulations where TAMRA is within 10 Å (quenched) of the Qb-MBP exposed intrinsic tryptophans (W101, W110 and W115) in the absence and presence of MBP. Data are from six independent simulations (R1-R6) over 72 μs (R1 – R6) considering the minimum distance between any of the tryptophans and TAMRA.

	No MBP	With MBP
R1	75.2	0
R2	77.6	0
R3	72.4	0.4
R4	64.2	0
R5	95.6	0
R6	38.9	0
Av ± std	**70.7 ± 18.7**	**0.1 ± 0.2**

Table S2 Percentage time in Qb-Lys where TAMRA is being quenched by any of the exposed intrinsic tryptophans in the absence and presence of antigen from MD simulations. Data from the six independent simulations over 72 μs (R1 – R6) considering the minimum distance between any of the tryptophans and TAMRA.

WT	No Lys	With Lys
R1	26.1	5.3
R2	19.5	8.5
R3	14.1	0
R4	30.6	16.2
R5	58.2	5.4
R6	75.2	0
Av ± std	**37.3 ± 24.1**	**5.9 ± 6.1**

Considering the reported accuracy of FoldX is 0.46 kcal/mol , mutations were considered:

highly stabilizing ($\Delta\Delta G < -1.84$ kcal/mol);
stabilizing (-1.84 kcal/mol $\leq \Delta\Delta G <$ -0.92 kcal/mol);
slightly stabilizing (-0.92 kcal/mol $\leq \Delta\Delta G <$ 0.46 kcal/mol);
neutral (-0.46 kcal/mol $< \Delta\Delta G \leq$ 0.46 kcal/mol);
slightly destabilizing (0.46 kcal/mol $< \Delta\Delta G \leq$ 0.92 kcal/mol);
destabilizing (0.92 kcal/mol $< \Delta\Delta G \leq$ 1.84 kcal/mol);
highly destabilizing ($\Delta\Delta G > 1.84$ kcal/mol).

Table S3 Solvent accessible surface area (ASA) of Qb-MBP and Qb-Lys in the absence and presence of their respective antigens. Average ± standard deviation calculated from 1 μs MD simulations of the apo and antigen-bound states. Maximum ASA of tryptophan = 259.0 Å .

**Residue**	**Apo state (Å^2)**	**Antigen-bound state (Å^2)**
5M14 scaffold		
W36	1.8 ± 1.6	3.3 ± 2.6
W101	165.3 ± 27.9	57.8 ± 9.0
W110	200.8 ± 22.4	66.9 ± 28.3
W115	79.4 ± 22.2	37.4 ± 11.2
1ZVH scaffold		

W36	3.2 ± 2.3	2.6 ± 2.1
W103	60.2 ± 11.7	6.1 ± 3.5
W115	75.4 ± 9.8	77.2 ± 14.9

Table S4 FoldX predictions of free energy changes (ΔΔG in kcal/mol) of protein stability and antigen binding due to W103Y and W115Y mutations in the Qb-Lys.

Mutation	Effect on protein stability	Effect on antigen binding
W103Y	3.5 (highly destabilising)	1.0 (affects binding)
W115Y	0.6 (slightly destabilising)	0.0 (neutral)

Table S5 Percentage time where TAMRA is being quenched by any of the exposed tryptophans in the absence and presence of antigen from MD simulations. Data from the six independent simulations over 72 μs (R1 – R6) considering the minimum distance between any of the tryptophans and TAMRA.

Y27W

	No Lys	With Lys
R1	35.9	10.1
R2	42.4	13.0
R3	53.0	0
R4	36.8	9.5
R5	42.0	0
R6	61.8	0.4
**Av ± std**	**45.3 ± 10.1**	**5.5 ± 6.0**

Y104W

	No Lys	With Lys
R1	31.7	2.6
R2	58.1	0.7
R3	76.2	0
R4	20.1	16.2
R5	84.0	0
R6	38.5	0
**Av ± std**	**51.4 ± 25.5**	**3.3 ± 6.4**

Y110W

	No Lys	With Lys
R1	56.1	0
R2	80.7	20.2
R3	21.0	0.4
R4	46.8	3.1
R5	34.1	4.5
R6	25.8	0
**Av ± std**	**44.1 ± 22.2**	**4.7 ± 7.8**

Table S6 FoldX predictions of free energy changes (in kcal/mol) of protein stability and antigen binding due to Y27W, Y104W and Y110W mutations.

**Mutation**	**Effect on protein stability**	**Effect on antigen binding**
Y27W	0.54	-0.33
Y104W	0.21	0.41
Y110W	-0.13	1.34
